# Data is the New Gold!

**DOI:** 10.5005/jp-journals-10071-23173

**Published:** 2019-06

**Authors:** Ashit V Hegde

**Affiliations:** Department of Critical Care Medicine, PD Hinduja Hospital and Medical Research Centre, Mumbai, Maharashtra, India

## Abstract

**How to cite this article:** Hegde AV. Data is the New Gold! Indian J Crit Care Med 2019;23(6):246.

“Above all else show the data”

—Edward Tufte

It is said that Data is the new gold, in which case, Indian ICUs are a very large gold mine. Unfortunately, this gold has not been mined. There is hardly any study from an Indian ICU, which has attempted to systematically record and analyze the data of admitted patients over several years.

The publication of “The STRIDE study” in this issue of the IJCCM is therefore very heartening.^1^ A total of 18940 patients were studied over 12 years, by all means a formidable task and the authors deserve to be congratulated.

Manual collection of data can be a tedious and daunting process. This study demonstrates that the CHITRA software greatly eases the collection of data and probably makes the data more accurate.

This study does have its limitations, chiefly—the inaccuracy of the admission diagnosis. Future studies should make all attempts to rectify this shortcoming. The results of this study are obviously useful to the Institution where it was performed.

However, sceptics might question the practical value and relevance of this study to intensivists practicing outside of the study institution. Admittedly the results from this study can not be considered to be representative of ICU's across the country. Yet the study, despite its limitations –is a very significant one. It clearly demonstrates that data collection and analysis (especially using the CHITRA software) is not such a challenging task.

This study should serve as an impetus to other ICUs to gather their own data. When data obtained from several ICUs is pooled, a clearer picture of the state of affairs in ICUs across our country will emerge. It might help ICUs compare their performance with those of other similar ICUs and might help improve the overall standards.

Such studies have public health benefits as well. A study of the trends in admission diagnoses might allow better allocation of resources for preventive health in areas where it is most needed.

Manu Varma and Sriram Sampath are the pioneering prospectors, it is time other ICUs in our country join this Gold Rush.
